# The long‐term efficacy of intra‐cervical lymphatic immunotherapy on adults with allergic rhinitis: A randomized controlled study

**DOI:** 10.1002/clt2.12341

**Published:** 2024-02-11

**Authors:** Yang Qin, Weijun Huang, Rui Zheng, Qixing Wang, Qingqing Yu, Yin Li, Kai Wang, Jun Tang

**Affiliations:** ^1^ The First Clinical Medical College Guangdong Medical University Zhanjiang China; ^2^ The Department of Otolaryngology First People's Hospital of Foshan Foshan China; ^3^ The Department of Otolaryngology Third Affiliated Hospital of Sun Y at‐sen University Guangzhou China; ^4^ The Zhu Hai Campus of Zunyi Medical University Zhuhai China

**Keywords:** allergens, allergic rhinitis, immunotherapy

## Abstract

**Background:**

The efficacy and safety of the novel immunotherapy method, intra‐cervical lymphatic immunotherapy (ICLIT), need to be investigated. Comparing it with subcutaneous immunotherapy (SCIT), we clarified the long‐term efficacy and safety of intra‐cervical lymphatic immunotherapy on allergic rhinitis (AR), and investigated the improvement of clinical efficacy of the booster injection at 1 year after ICLIT treatment.

**Methods:**

Ninety adult patients with dust mite allergy were randomly divided into 3 groups: 30 in the SCIT group, 30 in the ILCLIT group, and 30 in ICLIT booster group. Changes in total symptom score (TSS), nasal symptom score (TNSS), ocular symptom score (TOSS) and total medication score (TMS) were evaluated in the three groups. Adverse reactions were recorded, and serum dust mite specific IgE (sIgE) and specific IgG4 were assessed in the ICLIT group and ICLIT booster group.

**Results:**

TSS, TNSS, TOSS, and TMS scores were significantly lower in the three groups at 36 months after treatment (*p* < 0. 05). And at 36 months the ICLIT‐booster group showed results similar to SCIT and superior to ICLIT (*p* < 0. 05). Serum specific IgE decreased in all three groups at 12 and 36 months after treatment, *p* < 0.01. The ICLIT group and the ICLIT booster group showed a significant increase in sIgG4, *p* < 0.01. None of the patients in the three groups had any serious systemic adverse effects during the 3‐year follow‐up.

**Conclusion:**

The ICLIT treatment is effective and safe on AR. One booster injection of allergens at 1 year can greatly improve its long‐term efficacy.

**Trial Registry:**

Clinical trial registration number: ChiCTR1800017130.

## 1 BACKGROUND

Allergic rhinitis (AR) is an IgE‐mediated chronic inflammatory disease of the nasal mucosa that affects more than 500 million people in many countries. Allergic rhinitis can lead to reducing ability to work and is often associated with comorbid asthma, sleep problems, impaired quality of life, fatigue and emotional effects, in addition to the well‐known rhinitis and conjunctivitis.^1^ Allergen‐specific immunotherapy (AIT) can reduce the symptoms of AR and alter the course of the disease by targeting its cause.^2^, ^3^, ^4^, ^5^

In 2008, the Senti first reported a clinical trial of inguinal lymph node injection immunotherapy (ILIT).^6^ Since 2008, clinical studies on ILIT for the treatment of AR have been reported overseas and have demonstrated that the efficacy, safety and compliance of ILIT treatment regimens are higher than those of AIT.^7^, ^8^, ^9^, ^10^, ^11^, ^12^ Most of the allergens used in ILIT treatment are pollen and birch; therefore, there are few reports on ILIT treatment related to dust mite allergy. The allergens of AR vary in different areas of China, and the main allergen in the southern region is dust mite.^13^ There is a lack of reports on ILIT treatment protocols related to dust mite allergy, and the operation of ILIT treatment is inconvenient and exposes patient privacy.

Therefore, our team carried out the first ultrasound‐guided intra‐cervical lymphatic immunotherapy (ICLIT), and in 2019 we reported that ICLIT achieved better short‐term results and safety in the treatment of adults AR with dust mite allergies, which also significantly shortened treatment cycles, reduced treatment costs, and increased patient compliance.^14^ In 2023, we reported that ICLIT achieved better long‐term results.^15^ However, there is still a lack of clinical data to support its long‐term efficacy compared with SCIT and booster injections. Therefore, this study was conducted to clarify the long‐term efficacy and safety of intra‐cervical lymphatic immunotherapy on adult AR by comparing it with subcutaneous immunotherapy and to investigate the improvement of clinical efficacy of booster injections after 1 year of ICLIT treatment.

## 2 METHODS

### 2.1 Trial design

**FIGURE 1:**
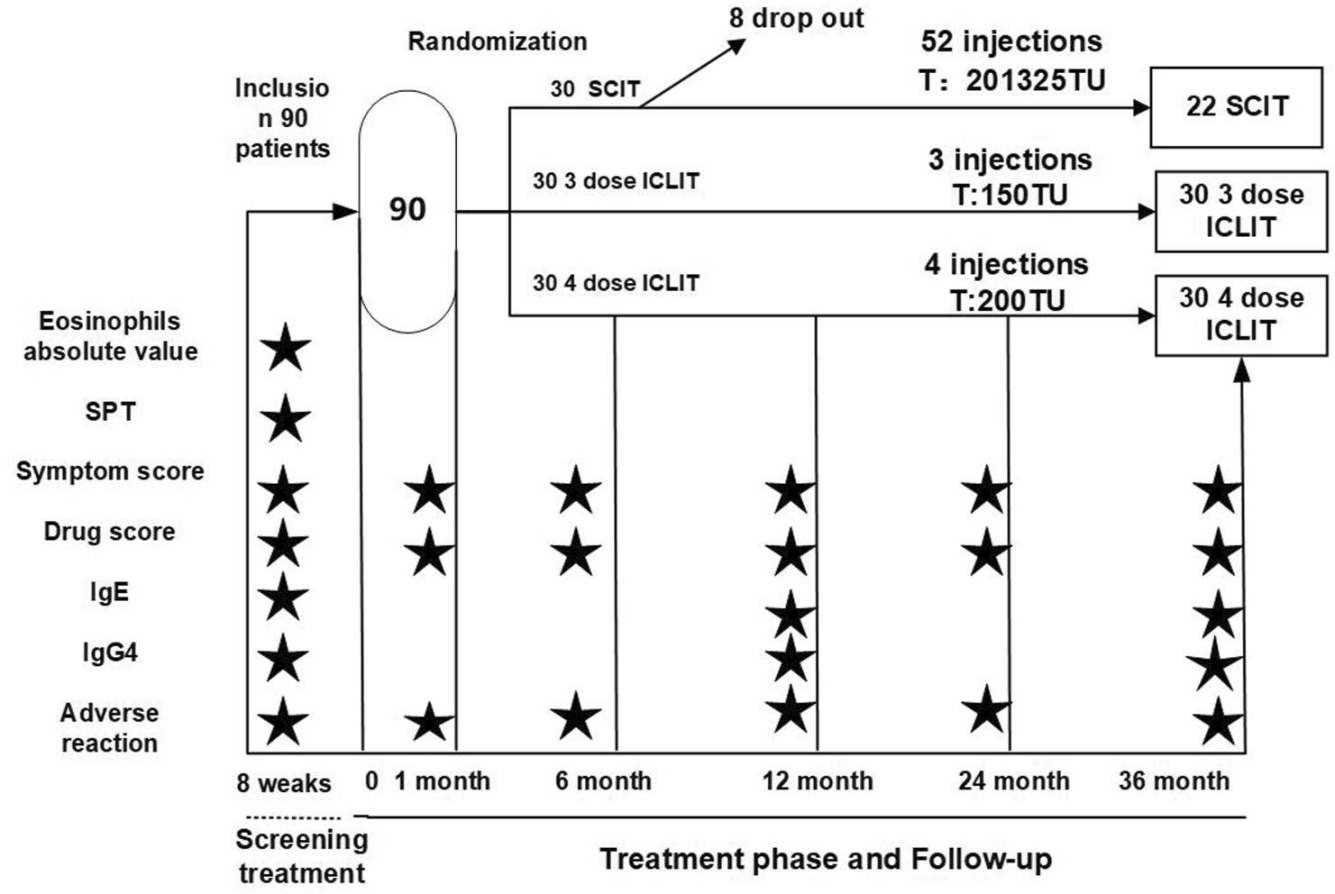
Trial design.The trial included 1 pre‐treatment screening, 3 treatments and I placebo in the ICLIT group, 4 treatments in the ICLIT booster group, 52 treatments in the SCIT group, and 5 follow‐up visits within 36 months, with IgE and IgG4 levels assessed before treatment and at 12 and 36 months. ★ data were collected at this point. ICLIT, intra‐cervical lymphatic immunotherapy; SCIT, subcutaneous immunotherapy.

The study design was a controlled randomized trial with 3 subgroups, the SCIT group, the ICLIT group, and the ICLIT booster injection group, as shown in Figure 1. The patients in ICLIT group and the ICLIT booster injection group did not know which group they were in until after 3 years. All study participants (or family members) signed a written informed consent. The study protocol was reviewed and approved by the Medical Ethics Committee of Foshan First People's Hospital (Ethics Research 2018 No. 10). Clinical trial registration number: ChiCTR1800017130.

### 2.2 Study population

A total of 90 study participants were recruited at the ENT outpatient clinic of Foshan First People's Hospital during 2018.02–2018.12. Patients who met the inclusion criteria were between 18 and 65 years of age and had a history of allergic rhino conjunctivitis due to dust mites according to ARIA diagnostic criteria, with at least two of four main nasal symptoms (nasal itching, sneezing, rhinorrhea and nasal congestion), with or without two ocular symptoms (ocular itching and lacrimation).^16^ Allergy was confirmed by Skin Prick Test (SPT) dust mite positivity (Soluprick Mites Allergens Prick Solution, 10 HEP, ALK‐Abello A/S, Horsholm Denmak, JS20210027) (there were Dermatophagoides pteronyssinus and Dermatophagoides farinae in the extract) and/or serum allergen dust mite specific IgE levels. Children were considered allergic to allergens by an allergen/histamine (A/H) ratio of ≥1 in the wheal diameter or serum level of allergen dust mite specific IgE ≥0.35 IU/mL. Exclusion criteria included Pregnancy or breastfeeding, pregnancy planning, autoimmune disease, vascular disease, asthma, nasal polyps, chronic obstructive and restrictive lung disease, cancer, major metabolic disease, alcohol or drug abuse, psychiatric disorders, or patients who may be unable to cooperate in completing treatment.

### 2.3 Treatment design

ICLIT group and ICLIT booster injection group: the study participants received the first 0.1 mL of concentration level 2, 500TU/mL (50TU per dose) Aroger's standardized dust mite allergen extract (Novo‐Helisen‐Depot, Allergopharma GmbH & Co. KG, Reinbek, Germany, JS20190020) (there was aluminum hydroxide in the extract) injections, while the second and third identical injections were given on day 28 (4 weeks) and day 56 (8 weeks). All three injections were performed on the same side; the injections were used by a 7‐gauge needle in a superficial zone II or III cervical lymph node (approximately 0.5–0.8 cm in size) identified by ultrasound guidance under strict aseptic manipulation. To prevent drug injection into the blood vessels, back‐drawing was performed before each injection. The patient's vital signs, peak expiratory flow, and local and/or systemic adverse reactions at the injection site were monitored in the hospital for 1 h after each injection. If adverse reactions occurred, then appropriate therapeutic measures were given according to the adverse reaction grading criteria.^17^ Patients (or family members) were instructed to record and report the specifics of all subsequent reactions within the next 24 h. At approximately 3 months (approximately 1 month after the last injection), 6 months, 12 months, 24 months, and 36 months after treatment, patients were asked to answer a questionnaire related to the first visit for comparison with the previous visit, with blood samples collected at 12 and 36 months to assess changes in serological parameters. ICLIT group will be treated with 0.1 mL normal saline as a placebo. The ICLIT booster group was treated with 0.1 mL (50 TU) of standardized dust mite allergen extract after 1 year of ICLIT treatment. And Blood samples taken at 1 year for relevant testing occurred at 1 week after booster injection. SCIT group: Subcutaneous immunotherapy was used as the control group for the study. The injections were administered in 2 phases, namely the initiation phase (dose escalation) and the maintenance phase (dose maintenance). The starting dose of 50 TU/mL of allergen (Novo‐Helisen‐Depot, Allergopharma GmbH & Co. KG, Reinbek, Germany, JS20190020) (there was aluminum hydroxide in the extract) injection was administered once a week for 15 weeks as follows: 0.1, 0.2, 0.4, and 0.8 mL of 50 TU/mL (concentration level 1) of allergen injection for the first 12 weeks, 0.1, 0.2, 0.4, and 0.8 mL of 500 TU/mL (concentration level 2) of allergen injection, and 0.1, 0.2, 0.4, and 0.8 mL of 5000 TU/mL (concentration level 3) of allergen injection, 5000TU/mL (concentration level3) allergen injection 0.1, 0.2, 0.4, 0.6 mL; and 5000TU/mL (concentration level 3) allergen injection 0.8, 1.0, 1.0 mL at weeks 13, 14 and 15, respectively. After 15 weeks, the dose maintenance phase was reached: a dose of 5000 TU/mL (concentration level 3) allergen injection 1.0 mL was maintained at an interval of 4 weeks between injections, and the total duration of treatment was 3 years. SCIT was evaluated before and after treatment in the same way as ICLIT.

### 2.4 Randomization

Ninety patients were randomized by a random number table. Starting from any random number in the random table, 90 random numbers were selected in order. The random numbers were sorted according to their size. Serial numbers 1–30 were divided into ICLIT group, 32–60 into ICLIT booster needle group, and 61–90 into SCIT group. The software of SPSS 26.0 was used.

### 2.5 Assessment methods

#### 2.5.1 Symptom scoring

Changes in nasal as well as ocular allergy symptoms associated with dust mites were scored individually using a visual analog scale with a range of 0–10 (0 for the mildest symptoms and 10 for the most severe symptoms). The total nasal symptom score (TNSS) and the total ocular symptom (TOSS) score were also added to produce a total symptom score (TSS) for each patient (0–60).

#### 2.5.2 Drug score

All patients were provided with rescue medications such as oral and topical antihistamines (nasal or ophthalmic), nasal glucocorticoids, and oral glucocorticoids. The principles of rescue drug use^18^: (1) oral or topical antihistamines/anti‐leukotriene drugs; (2) if symptoms could not be controlled, nasal glucocorticoids were added; (3) if the above did not control the symptoms of rhinitis, then oral glucocorticoids were added. According to the guidelines for the diagnosis and treatment of AR (2022, revised),^19^ the use of rescue medications associated with allergic symptoms was scored as 1 point/day for nasal, ophthalmic and/or oral antihistamines, 2 points/day for nasal glucocorticoids, and 3 points/day for oral glucocorticoids, and all medication scores were summed to give a total medication score (TMS).

#### 2.5.3 Adverse reactions

The safety of each treatment was assessed using a safety scale to record local adverse reactions and systemic adverse reactions in all patients during the treatment period. We recorded early adverse events during observations 1 h after each injection. Participants reported late adverse events by follow‐up 1 week after each injection. Local adverse reactions usually subsided spontaneously within 24 h. Systemic adverse reactions were classified in^19^: mild systemic reactions (local urticaria, rhinitis and mild asthma), moderate systemic reactions (slow onset, generalized urticaria and/or moderate asthma), severe systemic reactions (rapid onset, generalized urticaria or muscle angioedema and/or severe asthma) and anaphylaxis (rapid onset of pruritus, erythema, generalized urticaria, wheezing, asthma attacks, hypotension, etc.).

#### 2.5.4 Serological evaluation

The evaluation was performed by comparing the changes in circulating immunoglobulin (serum dust mite specific IgE) and serum specific IgG4 levels before treatment (baseline), at 12 months and at 36 months, by drawing peripheral venous blood (fasting), centrifuging the serum at 3500 r/min for 15 min at room temperature and storing it at −80°C for testing. Serum dust mite specific IgE was detected using a Thermo Fisher 250 automatic fluorescence immunoassay analyzer and ImmunoCAP Specific IgE enzyme secondary antibody kit, and the allergen criteria for detectionwere dust mite positive and the rest of the allergens negative. The results were divided into 0–6 levels according to IgE concentration. Level 0 is <0.35 kU/L; Level 1 ≥ 0.35 kU/L; Level 2 ≥ 0.70 kU/L; Level 3 ≥ 3.5 kU/L; Level 4 ≥ 17.5 kU/L; Grade 5 ≥ 50 kU/L; Level 6 ≥ 100 kU/L. Grade 0 indicates an allergy (−), grades 1–6 indicate varying degrees of allergy, and grade 1 and above is considered positive. Serum specific IgG4 antibodies test kit and House dust mite component specific IgG4 antibodies test kit were used. Results ≥155 U/ml, indicating that sIgG4 antibody of dust mite binds to dust mite antigen, and patients may produce an immune response to exposure to dust mite. The result was <155 U/ml, indicating that the concentration of sIgG4 in dust mites was undetectable or very low.

#### 2.5.5 Skin prick test

SPTs were administered to patients prior to treatment with nine common airborne allergens (ALK‐Ab) including dust mites Dermatophagoides pteronyssinus, Dermatophagoides farinae, animal allergens (cat hair, dog hair), weeds, trees, tropical mites, cockroaches, and mold II, on the palmar side of the forearm, with saline buffer as a negative control and histamine chloride (10 mg/mL) as a positive control. The allergen‐produced wheal is normally compared to the wheal produced by the negative control. A SPT is considered positive if the wheal diameter is 3 mm larger than the negative control within 20 min.^20^ The reaction intensity of SPT can be evaluated by skinindex (SI), which measures the largest wheal diameter and the diameter perpendicular to this one, respectively, in mm. The ratio of the average diameter of the two is SI, which is divided into 4 grades: + is 0.3 ≤ SI < 0.5; ++ is 0.5 ≤ SI < 1.0; +++ is 1.0 ≤ SI < 2.0; ++++ indicates that SI is ≥ 2.0.^19^

#### 2.5.6 Efficacy assessment

Efficacy was based on the guidelines for diagnosis and treatment of AR of the Rhinology Group of the Chinese Society of Otolaryngology, Head and Neck Surgery Branch, Chinese Journal of Otolaryngology, Head and Neck Surgery Editorial Committee^19^: (total score before treatment ‐ total score after treatment)/total score before treatment × 100%, >65% was judged as significant, 25%–65% as effective, ≤25% as ineffective. The effective rate = (number of effective cases + number of effective cases)/total number of patients × 100%.

### 2.6 Statistical analysis

Data were analyzed in this study using GraphPad Prism 9.0 (San Diego, CA, USA), and data distributions were assessed using the D'Agostino and Pearson integrated normality tests. Parametric paired data were analyzed with paired *t*‐tests, while nonparametric data were analyzed with Wilcoxon paired signed bilateral rank tests. For unpaired observations, unpaired *t*‐tests or Mann‐Whitney tests were used. Categorical variable data were expressed as frequencies, and data were compared using Fisher Exact Test and Pearson Chi‐Square to assess the amount of change in allergen type and SPT results before and after treatment. *p* values less than or equal to 0.05 were considered to be statistically significant.

## 3 RESULTS

### 3.1 Baseline information

Table 1 summarizes the baseline characteristics of the study subjects, and the mean age of patients in the three groups was 33.27 ± 9.42 years in the SCIT group, 31.13 ± 8.74 years in the ICLIT group, and 35.17 ± 10.89 in the ICLIT‐enhanced group (*p* > 0.05). Eight female participants in the SCIT group (36.4%), 12 females in the ICLIT group (40%) and 16 (53.3%) females in the ICLIT‐enhanced group. The baseline characteristics of the three groups were similar in terms of absolute eosinophil values, symptom scores, and allergen‐specific IgE (Table 1). All three groups of patients displayed house dust mite (Derp) or dust mite (Derf) allergy.

**TABLE 1:** Baseline characteristics by treatment group.

Characteristics	SCIT	3 dose ICILT	4 dose ICILT
Enrolled subjects	22	30	30
Age at study start (y)	33.27 ± 9.42	31.13 ± 8.74	35.17 ± 10.89
Gender			
Female (%)	8 (36.4)	12 (40)	16 (53.3)
Male (%)	14 (63.6)	18 (60)	14 (46.7)
Eosinophils absolute value, mean ± SD(×10^9^/L)	0.34 ± 0.22	0.36 ± 0.22	0.32 ± 0.17
Total symptom score, mean ± SD	31.68 ± 3.14	32.43 ± 2.03	32.57 ± 3.10
Allergen‐total IgE(KU/L)	292.5 (39.3–555)	184.5 (134–375.5)	167 (75.0–359.4)
Allergen‐specific IgE(KU/L)			
Derp	14.4 (5.85–45.97)	15.15 (1.14–35.05)	13.32 (2.11–29.3)
Derf	17.95 (4.83–43.82)	13.25 (1.15–33.63)	16.65 (2.24–31.53)
SPT (Derp)			
−	2 (9.1%)	4 (13.3%)	1 (3.3%)
+	1 (4.5%)	0 (0.0%)	1 (3.3%)
2+	9 (41.0%)	10 (33.3%)	8 (26.7%)
3+	5 (22.7%)	12(40.0%)	15 (50.0%)
4+	5 (22.7%)	4 (13.3%)	5(16.7%)
SPT (Derf)			
−	1 (4.5%)	4 (13.3%)	3 (10.0%)
+	0 (0.0%)	1 (3.3%)	2 (6.7%)
2+	12 (54.5%)	10 (33.3%)	9 (30.0%)
3+	7 (31.8%)	12 (40.0%)	15 (50.0%)
4+	2 (9.1%)	3 (10.0%)	1 (3.3%)

*Note*: A total of 22 cases participated in SCIT group, 30 cases in ICLIT group and 30 cases in ICLIT booster group. Numerical parameters conforming to normal distribution are expressed as mean (standard deviation), non‐conforming to normal distribution or median (quartile), and other parameters are expressed as frequency. Skin index: Normal: “0” = “−”; Level 1: “+” = SI < 0.5; Level 2: “2+” = 0.5 ≤ SI < 1.0; Level 3: “3+” = 1.0 ≤ SI < 2.0; Level 4: “4+” = 2.0 ≤ SI. SPT: Skin Prick Test.

Abbreviations: Derf, Dermatophagoides farinaespecific; Derp, Dermatophagoides pteronyssinus; SD, sample standard deviation.

### 3.2 Adherence

**FIGURE 2:**
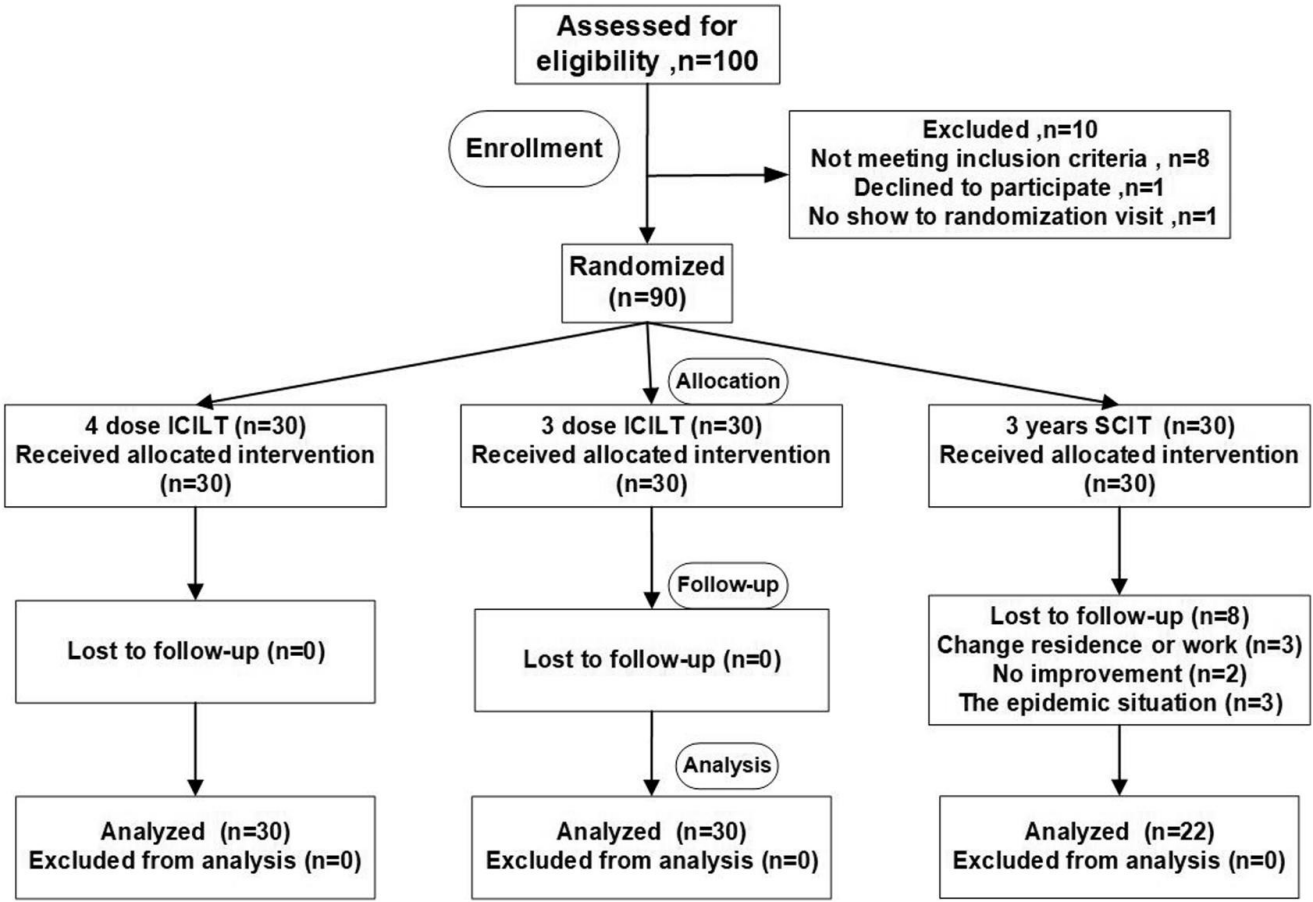
The flow of patients. A total of 100 rhinitis patients were assessed for eligibility. 10 cases did not meet the inclusion criteria. A total of 90 patients with dust mite allergy were assigned to the SCIT group, the ICLIT group and the ICLIT booster group. Of the 30 patients in the SCIT group, 22 completed the 3‐year treatment and completed the long‐term follow‐up; Of the 30 patients assigned to the ICLIT group, 30 completed a 3‐dose injection and completed a 3‐year long‐term follow‐up. Of the 30 patients assigned to the ICLIT booster group, 30 completed the 4‐needle injection and completed a 3‐year long‐term follow‐up. ICLIT, intra‐cervical lymphatic immunotherapy; SCIT, subcutaneous immunotherapy.

As shown in Figure 2. A total of 90 dust mite allergic patients eventually met the inclusion criteria, and the 90 participants were randomly assigned to the SCIT, ICLIT, and ICLIT booster injection groups according to the route of administration of immunotherapy (Figure 2). Accordingly, 30 patients were divided into ICLIT and ICLIT booster injection groups, and all patients completed treatment. 30 patients were divided into SCIT group, 22 patients completed treatment, and 8 patients did not complete treatment (for drop‐out reasons, see Figure 2). Finally, 30 patients in the ICLIT group, 30 patients in the ICLIT booster injection group, and 22 patients in the SCIT group were compared for data analysis.

### 3.3 ICLIT treatment was effective

The baseline data of patients in the ICLIT group were analyzed as shown in Table 2. The short‐term efficacy of cervical lymph node immunotherapy in the ICLIT group was positive. Compared with the baseline, the overall efficacy of TSS in the ICLIT group at 12 months, 24 and 36 months was 50%, 41% and 31%, and the overall efficacy of TNSS was 52%, 42% and 32%, respectively. The overall efficacy of TOSS was 44%, 33%, and 27%, respectively, and the TMS decreased to 62%, 48%, and 36%, respectively, indicating that the efficacy of ICLIT patients gradually deteriorated 1 year after treatment.

**TABLE 2:** TSS, TNSS, TOSS and total medication score in SCIT group, ICLIT group and ICLIT booster group.

Group		Baseline	3 months	6 months	12 months	24 months	36 months
SCIT	TSS mean (SD)	31.68 (3.14)	23.73 (4.44)	20.14 (4.38)	17.23 (6.05)	14.55 (7.22)	12.27 (10.43)
	TNSS mean (SD)	25.32 (3.41)	19.55 (3.98)	15.73 (3.49)	14.55 (5.36)	12.95 (5.76)	10.64 (8.44)
	TOSS mean (SD)	6.36 (2.17)	4.18 (2.11)	4.41 (1.44)	2.68 (1.78)	1.59 (1.97)	1.64 (0.46)
	TMS mean (SD)	3.05 (0.84)	1.68 (0.95)	1.45 (1.01)	0.91 (1.02)	0.91 (0.92)	0.86 (1.08)
ICLIT	TSS mean (SD)	32.43 (2.03)	25.17 (4.77)	21.10 (6.88)	16.10 (9.36)	19.13 (10.06)	22.47 (11.85)
	TNSS mean (SD)	27.30 (2.88)	21.10 (3.95)	17.60 (5.59)	13.23 (7.19)	15.70 (8.02)	18.70 (9.65)
	TOSS mean (SD)	5.13 (3.23)	4.07 (2.74)	3.50 (2.71)	2.87 (3.01)	3.43 (2.86)	3.77 (3.17)
	TMS mean (SD)	2.13 (0.97)	1.50 (0.86)	1.07 (0.69)	0.80 (1.00)	1.10 (0.92)	1.37 (1.22)
ICLIT booster	TSS mean (SD)	32.57 (3.10)	24.67 (5.63)	21.13 (7.21)	18.03 (8.92)	17.30 (10.14)	16.77 (11.46)
	TNSS mean (SD)	26.27 (4.16)	20.27 (5.43)	17.63 (5.90)	15.23 (7.00)	14.80 (8.12)	14.20 (9.32)
	TOSS mean (SD)	6.30 (3.34)	4.40 (2.72)	3.59 (2.54)	2.80 (2.78)	2.50 (2.92)	2.56 (3.13)
	TMS mean (SD)	1.93 (1.14)	1.33 (0.96)	1.10 (0.92)	1.13 (1.28)	0.90 (1.06)	0.83 (1.02)

*Note*: 3,6,12,24,36 months means each time period of follow‐up after treatment.

Abbreviations: TMS, Total medication score; TNSS, Total nasal symptom scores; TOSS, Total ocular scoring system; TSS, Total symptom score.

### 3.4 The long‐term efficacy of ICLIT treatment was inferior to that of SCIT

**FIGURE 3:**
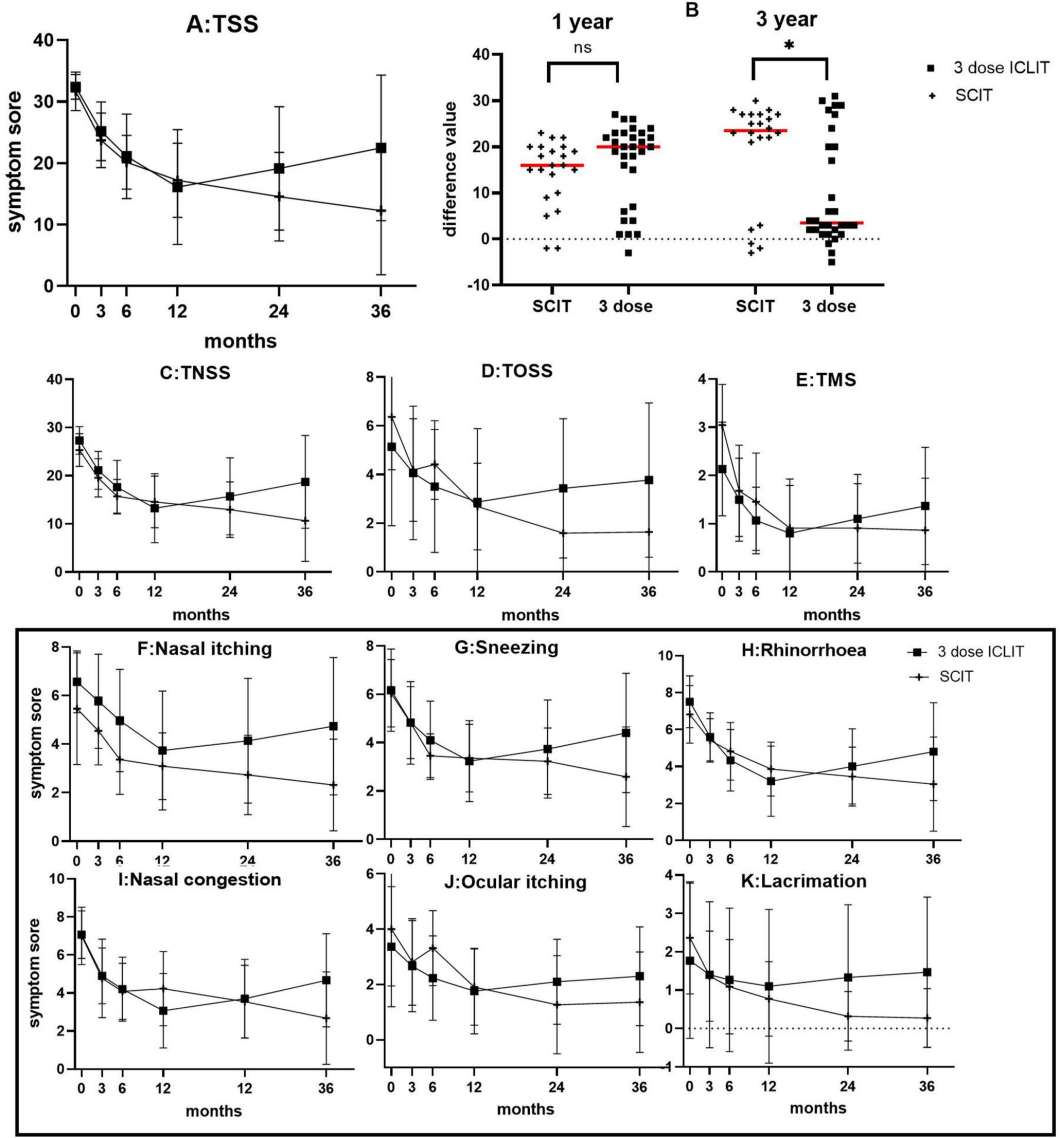
(A) TSS scores of ICLIT group and SCIT group for each time period. (B) There was no significant difference in the change of TSS between baseline and 12 months (ns means *p* > 0.05), and there was a significant difference in 36 months (* means *p* < 0.05). (C–E) TNSS, TOSS and TMS of ICLIT group and SCIT group for each time period. (F–K) Scores of nasal and ocular symptoms in ICLIT group and SCIT group. ICLIT, intra‐cervical lymphatic immunotherapy; SCIT, subcutaneous immunotherapy; TNSS, Total nasal symptom scores; TOSS, Total ocular scoring system; TSS, Total symptom score.

There was no significant difference at the baseline between ICLIT group and SCIT groups (*p* > 0.05). The scores of TSS (Figure 3A), TNSS (Figure 3C) and TOSS (Figure 3D) in ICLIT group and SCIT groups were similar in 3 months, 6 and 12 months. However, at 24 and 36 months of treatment, the scores of TSS, TNSS and TOSS in ICLIT group were higher than those in SCIT group. The change in TSS between the two groups (pre‐treatment score ‐ post‐treatment score) was further analyzed, and the changes in the ICLIT group and the SCIT group at 12 months of treatment were 16.33 (1.64) and 14.45 (1.55), respectively, and there was no difference between the two groups (*p* > 0.05). The change of 36 months of treatment was 9.97 (2.15) and 19.41 (2.38), respectively, and there was a difference between the two groups (*p* < 0.05) (Figure 3B), indicating that the long‐term therapeutic effect of ICLIT group was worse than that of SCIT group.

### 3.5 ICLIT booster injection improves long‐term outcome

**FIGURE 4:**
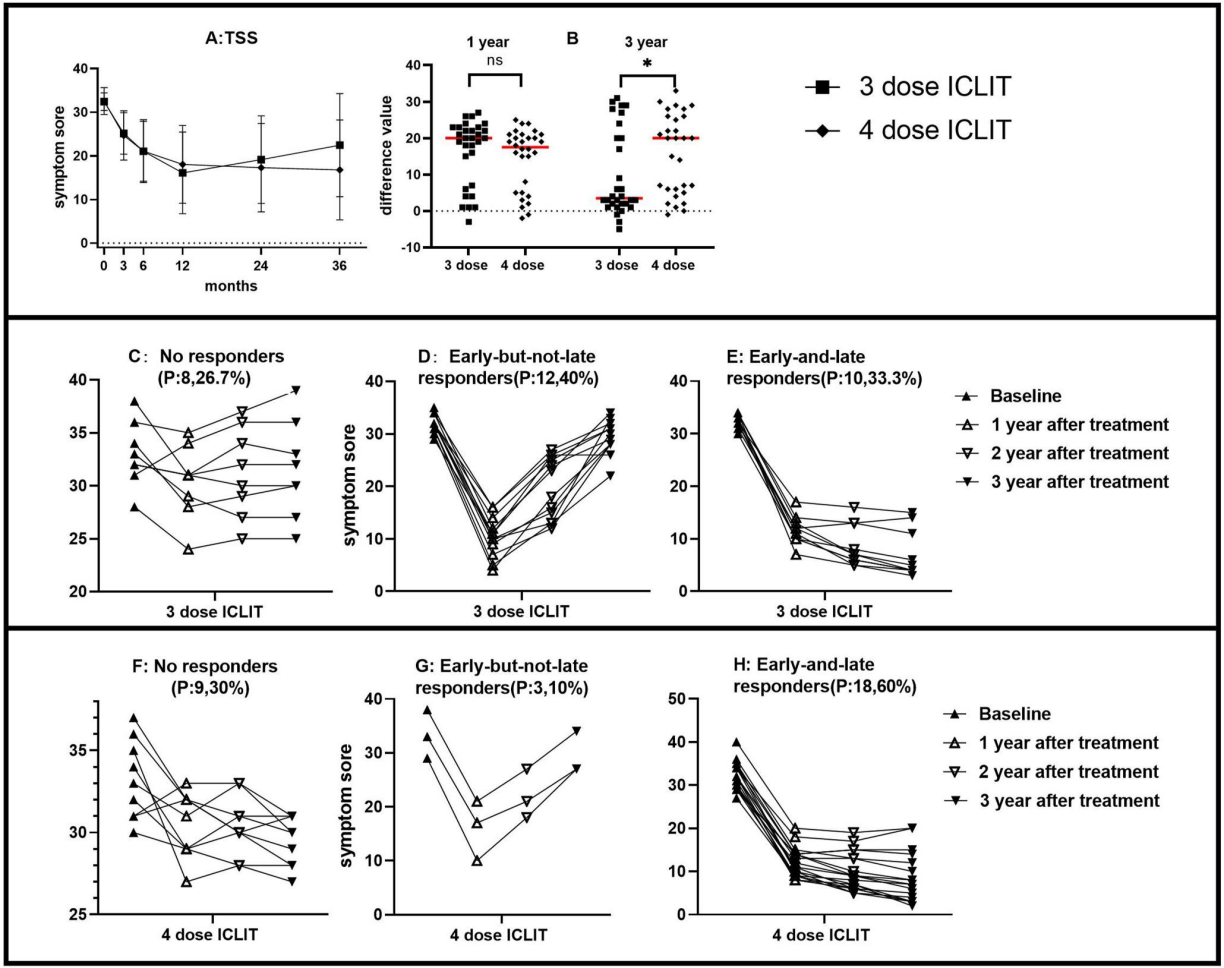
(A) TSS of ICLIT group and ICLIT booster group for each time period. (B) There was no significant difference in the change of TSS between baseline and 12 months (ns means *p* > 0.05), and there was a significant difference in 36 months (* means *p* < 0.05). (C and F) The number of No responders in ICLIT group and ICLIT booster group. (D and G) The number of early‐but‐not‐late responders in ICLIT group and ICLIT booster group. (E and H) The number of early‐and‐late responders in ICLIT group and ICLIT booster group. ICLIT, intra‐cervical lymphatic immunotherapy; SCIT, subcutaneous immunotherapy; TSS, Total symptom score.

Analysis of ICLIT group and ICLIT booster group: TSS in the ICLIT group and the ICLIT booster group continued to decrease at 3, 6 and 12 months of treatment (Table 2); however, TSS in the ICLIT group increased at 24 and 36 months of treatment compared with 12 months after treatment, while the ICLIT booster group continued to decrease (Figure 4A). The change in TSS in the two groups at 12 and 36 months of treatment compared with baseline was further compared and analyzed. The change between the two groups at 12 months was 16.33 (1.64) and 14.53 (1.53), respectively, with no significant difference (*p* > 0.05). The changes between the two groups at 36 months were 9.97 (2.15) and 15.80 (1.99). There was a difference (*p* < 0.05) (Figure 4B), which was statistically significant. Depending on whether the treatment is effective for 12 months (short term) and 36 months (long term). The patients were divided into three groups: No responders, early‐but‐not‐late responders, and early‐and‐late responders. In the ICLIT group, the first category consisted of 8 people, accounting for 26.7% (Figure 4C); The second category consisted of 12 people, accounting for 40% (Figure 4D). The third group consisted of 10 people, accounting for 33.3% (Figure 4E). In ICLIT booster group, the first category consists of 9 persons, accounting for 30% (Figure 4F); The second category consists of 3 people, accounting for 10% (Figure 4G); The third group consists of 18 people, accounting for 60% (Figure 4H).

### 3.6 The long‐term efficacy of ICLIT booster and SCIT was comparable

**FIGURE 5:**
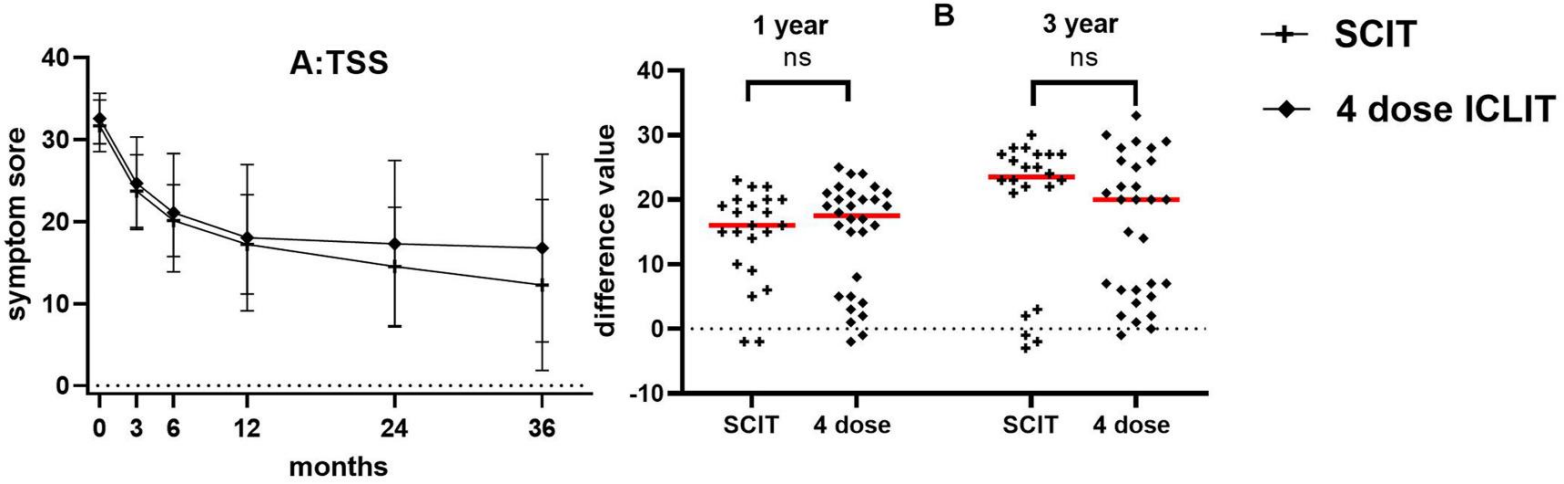
(A) TSS of SCIT group and ICLIT booster group for each time period. (B) There was no significant difference in the change of TSS between baseline and 12 months (ns means *p* > 0.05), and there was no significant difference in 36 months (ns means *p* > 0.05). ICLIT, intra‐cervical lymphatic immunotherapy; SCIT, subcutaneous immunotherapy; TSS, Total symptom score.

Analysis of ICLIT booster group and SCIT group: TSS of ICLIT booster group and SCIT group continued to decrease at 3, 6, 12, 24 and 36 months of treatment (Figure 5A). Further analysis of the difference between the two groups, the change of TSS at baseline and 12 or 36 months showed no significant difference between the two groups (*p* > 0.05) (Figure 5B).

### 3.7 IgE

**FIGURE 6:**
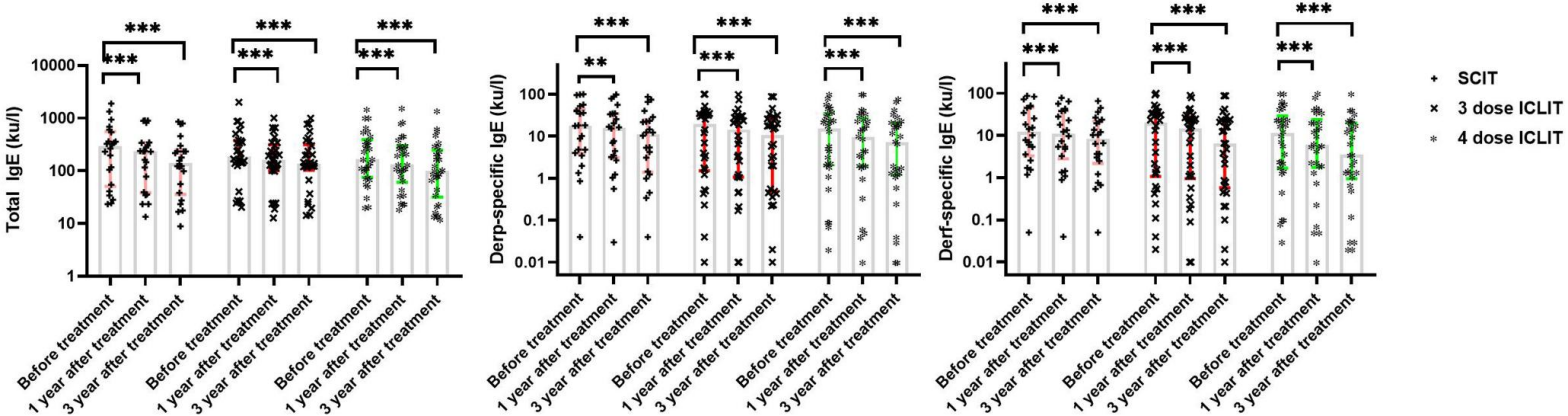
IgE changes in SCIT, ICLIT and ICLIT booster groups at post‐treatment and pre‐treatment (**p < 0.01; ***p < 0.001). ICLIT, intra‐cervical lymphatic immunotherapy; SCIT, subcutaneous immunotherapy.

Comparative analysis of the three groups showed that serum total IgE, house dust mite specific IgE and dust mite ‐ specific IgE decreased at 12 and 36 months of treatment compared with baseline(*p* < 0.01), and it was found that the decrease of patients in the three groups at 36 months of treatment was greater than that at 12 months of treatment (Figure 6).

### 3.8 IgG4

**FIGURE 7:**
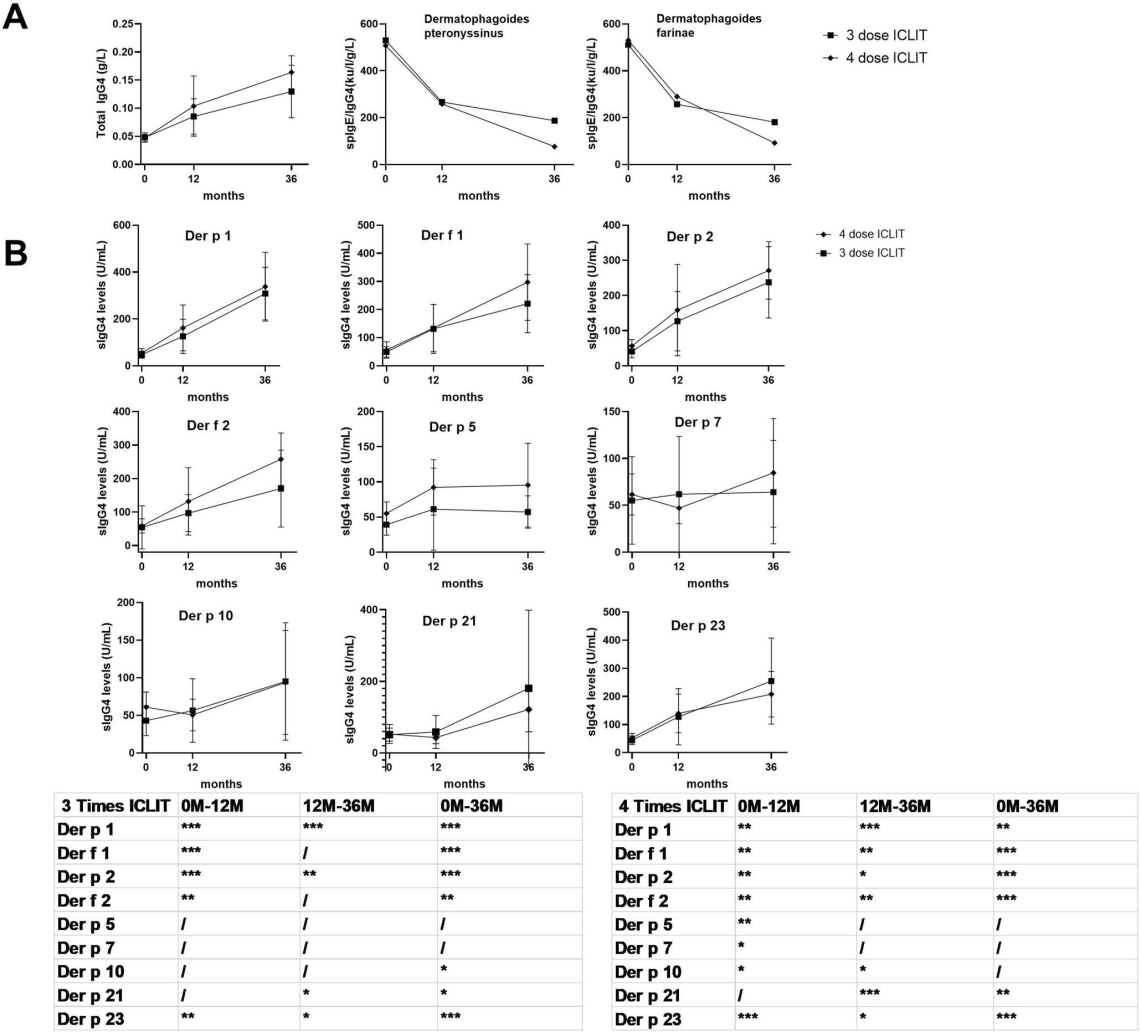
(A) The longitudinal changes in total IgG4 and the spIgE/IgG4 of dermatophagoides pteronyssinus and dermatophagoides farina. (B) Dust mite sIgG4 in ICLIT group and ICLIT booster group during 36 months of AIT and the *p* value of sIgG4 of ICLIT group and ICLIT booster group between 0 and 12 M, 12–36 M, 0–36 M. (/> 0.05; *p < 0.05; **p < 0.01; ***p < 0.001). Der f, Dermatophagoides farinaespecific; Der p, Dermatophagoides pteronyssinus; ICLIT, intra‐cervical lymphatic immunotherapy; SCIT, subcutaneous immunotherapy.

Compared with baseline, serum total IgG4 was significantly increased in ICLIT group and ICLIT booster group at 12 and 36 months *p* < 0.01. While some kinds of sIgG4 were significantly increased in both the groups at 12 and 36 months, respectively. *p* < 0.01 (Figure 7A,B).

### 3.9 Safety

Adverse reactions were triggered during treatment in three groups of patients (Table 3), Local adverse reactions were mainly pruritus, rash, redness, swelling, hard nodules and even necrosis at the injection site of allergen vaccine, 22 patients treated with SCIT had a total of 1144 injections and 162 (14.2%) adverse events, including 112 local mild‐to‐moderate adverse reactions (112 early and 0 late), 50 (4.4%) systemic mild‐to‐moderate adverse reactions (50 early and 0 late). All of the early systemic adverse events subsided within 24 h and no late symptoms required treatment, and the next treatment proceeded smoothly as scheduled. 90 injections were performed in 30 patients treated with ICLIT, and only 9 (10.0%) early mild local adverse events were occurred. 30 patients treated with ICLIT booster injections had a total of 120 injections, and only 8 (6.6%) early mild local adverse events (2 reactive lymph node enlargements, 3 itching around the puncture port, and 3 redness around the puncture port), and all of which were treated with oral antihistamines. No moderate to severe local or systemic adverse reactions were observed, and all patients were able to complete the entire program as planned.

**TABLE 3:** Adverse events associated with the intralymphatic injections.

	Order of severity	SCIT 22P (1144)		3 dose ICLIT 30P (90)		4 dose ICLIT 30P (120)	
		Early reactions	Late reactions	Early reactions	Late reactions	Early reactions	Late reactions
Local reactions							
Injection swelling	Mild	4P (39)	None	2P (3)	None	2P (2)	None
	Moderate	3P (12)		None		None	
	Severe	None		None		None	
Itch	Mild	4P (34)	None	2P (2)	None	2P (3)	None
	Moderate	3P (9)		None		None	
	Severe	None		None		None	
Redness	Mild	3P (13)	None	3P (4)	None	2P (3)	None
	Moderate	3P (5)		None		None	
	Severe	None		None		None	
Systemic reactions							
Abdominal pain or nausea	Mild	3P (4)	None	None	None	None	None
	Moderate	2P (3)					
	Severe	None					
Headache	Mild	2P (2)	None	None	None	None	None
	Moderate	None					
	Severe	None					
Urticaria and angioedema	Mild	3P (4)	None	None	None	None	None
	Moderate	2P (3)					
	Severe	None					
Pulmonary symptoms	Mild	2P (6)	None	None	None	None	None
	Moderate	None					
	Severe	None					
Eye or nasal symptoms	Mild	3P (18)	None	None	None	None	None
	Moderate	None					
	Severe	None					

*Note*: P: person. A total of 1144 injections were given in the SCIT group, 90 injections in the ICLIT group, and 120 injections in the ICLIT booster group. Mild systemic reactions (local urticaria, rhinitis and mild asthma), Moderate systemic reactions (slow onset, generalized urticaria and/or moderate asthma), Severe systemic reactions (rapid onset, generalized urticaria or muscle angioedema and/or severe asthma).

## 4 DISCUSSION

In recent years, AR has become a global health issue and is one of the allergic diseases that researchers focus on, and immunotherapy (AIT) is recommended as a first‐line treatment option in several domestic and international guidelines such as AAIR and EAACI.^4^, ^21^ A large number of studies have shown that its treatment regimen can prevent the occurrence of new allergies.^22^, ^23^ During the COVID‐19 pandemic, when patients did less outdoor activities and were less likely to visit the hospital, the ideal AIT regimen should achieve good clinical efficacy and mediate long‐term immune tolerance with a small dose in a short period of time.^24^ This study is the first 3‐year prospective randomized controlled trial to investigate the clinical efficacy and safety of intra‐cervical lymphatic immunotherapy (ICLIT) for the treatment of mite AR. Although this was a preliminary long‐term study based on a limited number of patients, ICLIT provided durable relief of nasal allergy symptoms and did not cause serious adverse events. In this study, 90 participants were enrolled, 60 participants were divided into ICLIT and ICLIT booster injection groups, and all patients completed treatment on time and were followed up for 3 years, with a dropout rate of 0%. 30 participants were divided into SCIT groups, 22 participants completed treatment, and 8 patients did not complete treatment and follow‐up, with a dropout rate of 26.7%. In 2008, the Senti et al.^6^ conducted a preliminary randomized controlled clinical trial of ILIT for rhinitis and found that even with very low doses of allergenic ILIT, efficacy was comparable to that of SCIT, and compliance was higher in ILIT patients than in SCIT. In 2013, an open double‐blind controlled clinical trial by Hylander et al.^9^ also confirmed higher compliance and acceptance of ILIT therapy than SCIT. In this study, the high dropout rate of SCIT was mainly due to the impact of the COVID‐19 pandemic, while ICLIT received significantly fewer injections and had significantly higher acceptance and compliance than SCIT during the COVID‐19 pandemic. SCIT and SLIT are currently used for AR. Disadvantages of SCIT include long‐term and the risk of systemic adverse events. SLIT has been developed to be more patient‐friendly, but the frequency and duration of treatment remain long. Since 2008, the Senti et al.^6^ first reported a clinical trial on ILIT for the treatment of AR and she concluded that the efficacy of ILIT was faster and safer than that of SCIT. In her randomized controlled trial, 165 patients were randomized to receive either SCIT (54 injections over 3 years) or ILIT (3 injections over 2 months) and she found that the ILIT group within 4 months immediately showed improvement in nasal symptoms and produced immune tolerance comparable to that of SCIT. Subsequently, articles on ILIT for rhinitis were reported. In 2012, Hylander et al.^9^ reported 15 patients randomized 1:1 to receive either active ILIT or placebo ILIT using standardized grass or birch pollen extracts, and found that active ILIT was associated with a significant improvement in seasonal symptoms after treatment compared with placebo; they also found that after nasal allergen provocation, active ILIT resulted in a significant reduction in nasal symptom scores. Another study in 2020, 21 patients were randomized to active or placebo ILIT for allergic rhinoconjunctivitis due to mountain cedar pollen. The results showed that in the active ILIT group there was persistent improvement in seasonal symptoms.^25^ In 2022, Ahlbeck et al.^26^ focused on the use of ILIT with one or two allergens to produce similar clinical responses in patients suffering from AR caused by birch and grass pollen. Three groups: one receiving three doses of birch and 5‐grass allergen extract, one receiving birch and placebo, and one receiving 5‐Grass and placebo. By ultrasound‐guided monthly injections into the inguinal lymph nodes, and after treatment with ILIT, they found that participants had fewer symptoms, reduced medication use, and improved quality of life after treatment with one or two allergens independently of each other during the annual birch and grass pollen season. These studies suggest that ILIT is a faster form of AIT that provides a long‐term reduction in AR symptoms equivalent to SCIT. In this study, ICLIT was used to treat adults with dust mite AR, and we observed a significant reduction in nasal eye symptom scores and a remarkable reduction in rescue medication dependence in both groups at 3 months, 6 months, 12 months, 24 and 36 months of treatment compared to pre‐treatment. After 24 and 36 months of treatment, ICLIT continued to maintain symptom improvement and continued reduction in rescue medication dependence, which is similar to the findings of Hylander and Ahlbeck. This indicates that the long‐term efficacy of ICLIT for adult dust mite AR is sustained and stable.

We conducted further short‐term and long‐term efficacy studies comparing the ICLIT group with the SCIT group. In the short‐term efficacy assessment at 1 year, we found that patients in the ICLIT and SCIT groups had comparable efficacy, and both groups produced consistent short‐term immune tolerance; In the long‐term efficacy assessment at 3 years, we observed that the long‐term efficacy of the ICLIT group was significantly lower than that of the SCIT group. The patients in ICLIT group could be divided into three cohort groups: No responders, early‐but‐not‐late responders, and early‐and‐late responders. There were 12 persons accounting for 40% called early‐but‐not‐late responders who had a good early stage response, but this was not maintained on the long term.. Similar situation occurred with conventional immunotherapy, and several scholars like the Yuan and Yang also analyzed its poor long‐term efficacy and they found it resulted from an inadequate course of treatment or low maintenance dose.^27^, ^28^ While ILIT has been carried out in relevant clinical trials, in 2021 Weinfeld et al.^29^ carried out a double‐blind placebo‐controlled randomized trial by administering ILIT pre‐season booster to AR patients with grass pollen allergy, and the findings confirmed that the booster improved the allergic reactions caused during the grass pollen season with lower side effects compared to placebo. In 2020, Skaarup et al.^10^ scholars published a double‐blind randomized placebo‐controlled trial on a 3‐year follow‐up, in which grass pollen rhinoconjunctivitis patients received 3 ILIT injections and 1 ILIT booster, 3 ILIT injections and 1 placebo booster, or 3 placebo injections and 1 placebo booster after 1 year. The findings indicated that ILIT significantly reduced grass pollen allergy symptoms and the use of rescue medication, with a significant effect in the first season after treatment and no additional effect of booster injections. In this study, the ICLIT booster injection group was performed more efficiently. We chose to inject the booster injection in the first year because in previous clinical follow‐up, we found that 1 year after ICLIT immunotherapy, 1/3 of the patients felt that the degree of symptom relief had decreased. Therefore, we designed to inject 0.1 mL of Aroger's standardized dust mite allergen extract once more a year after the initial ICLIT. After giving 1 ICLIT booster treatment, it was found that the number of patients of early‐but‐not‐late responders decreased significantly, while the number of patients of early‐and‐late responders increased. The long‐term effect of ICLIT booster group was consistent with that of the SCIT group. Therefore, a booster injections can improve the long‐term curative effect of patients. The immune tolerance of the body is related to the injection dose, the injection interval, and number of injections. For the group with poor curative effect, the possible cause is related to the increase in IgG4 production in immune tolerance. The additional dose given 1 year after treatment can increase the IgG4 production, but the specific mechanism still needs to be further explored. The Danish group recently showed that the efficacy of ILIT is directly related to the number of ‘correct’ intralymphatic injections. The relationship between injection success and efficacy was not focused on in our study, which we will refine in future studies.^30^

In various studies of conventional immunotherapy, it has been shown that immunotherapy restores the body's immune tolerance to allergens through multiple mechanisms, corrects immune imbalances, downregulates allergen‐specific T and B cell responses and results in decreased IgE production and increased IgG4 production.^31^ The latter cannot bind to complements, has limited affinity for Fcγ receptors, and most importantly, it competes with allergen‐specific IgE, thus preventing mast cell and basophil degranulation.^31^, ^32^, ^33^ The hallmark of successful SCIT treatment is demonstrated by an increase in serum‐specific IgE in the beginning, a slow decrease over time, and a decrease in serum‐specific IgE that favors an increase in IgG (especially serum‐specific IgG4).^1^, ^34^ Previous studies have shown that the production of allergen specific IgG4 reaction is considered to be a “protective" factor for AIT.^35^, ^36^ Despite the potential protective effect of IgG4, many studies have failed to demonstrate a correlation between IgG4 and clinical response to AIT.^37^, ^38^ Senti et al. found that serum IgE levels were significantly lower in patients 36 months after treatment, which was compared to who received subcutaneous treatment.^6^ In a 2017 article Lee et al.^39^ reported 11 patients with AR were treated with ILIT and found no significant changes in serum specific IgE and specific IgG4 in patients with animal dander allergy after 1 year of treatment, while a significant increase in serum IgG4 was found in patients with dust mite allergy after 1 year of treatment. The reason for this occurrence was considered to be more correlated with allergens. In a study after intralymphatic immunotherapy with recombinant MAT‐Feld1, a decrease in allergen‐specific IgE levels after 3 years of ILIT treatment and a decrease in allergen‐specific IgG4 levels after 1 year of ILIT treatment were reported.^40^ We found that in all three groups of patients, dust mites Dermatophagoides pteronyssinus and Dermatophagoides farinaespecific IgE were significantly lower than before treatment, whereas dust mites Dermatophagoides pteronyssinus and Dermatophagoides farinae specific IgG4 increased yearly after 1 year and 3 years, but no symptom improvement at 3 years in ICLIT‐no booster group, which is similar to the findings of Freiberger et al.^40^ However, there was little correlation between its increase and symptom improvement, which was similar to Lin Yang et al.^41^ In this study, there were no moderate to severe local adverse reactions or systemic adverse reactions, except for early local adverse reactions. The excellent safety profile of ICLIT compared with SCIT may be related to the fact that the allergen dose required for a single injection in the lymph nodes is approximately 1/100th of that required for a subcutaneous injection. In a preliminary study of intralymphatic immunotherapy, the initial dose of allergen was a 1000‐fold dilution of the maximal concentration of allergen extract for subcutaneous immunotherapy for house dust mite, cat and dog allergy, patients experienced 7 localized mild symptoms around the injection site, 4 experienced severe side effects in the form of generalized itching, urticaria, diarrhea or headache, and 2 suffered anaphylaxis.^39^ In a larger randomized clinical study of 63 patients, Hylander^25^ found only mild side effects. Similarly, in other placebo‐controlled trials, no serious systemic adverse reactions occurred during treatment. ICLIT not only had significant long‐term efficacy but also greatly increased safety, providing clinical support for our further promotion of this treatment.

In this study, due to relevant ethical issues, placebo was not used for comparison, which is an important limitation., we chose to compare it with traditional subcutaneous immunotherapy. In future studies, we hope to enroll volunteers to complete placebo‐controlled studies. The study was a single‐center prospective randomized controlled study and lacked large sample data. Therefore, future studies of ICLIT for AR will aim to validate multicenter large‐sample data, and the molecular mechanism of ICLIT‐induced immune tolerance deserves further investigation. ICLIT may be a new option in the future treatment of AR.

## 5 CONCLUSION

This study demonstrated for the first time that ICLIT treatment in adult AR has long‐term efficacy, induces immune tolerance, and is safe. Giving one booster injection of allergens 1 year after ICLIT treatment greatly improved its long‐term efficacy.

## AUTHOR CONTRIBUTIONS

Yang Qin wrote this manuscript and data analysis, Weijun Huang was responsible for patiently administering Intracervical lymphotic injections to each patient. Rui Zheng and Qixing Wang collected data and followed up patients, Qingqing Yu and Yin Li processed statistical data on the manuscript, Kai Wang independently completed the research design and concept, Jun Tang solved complex issues in the manuscript, and made final review and finalization. All authors listed have made a substantial, direct, and intellectual contribution to the work and approved it for publication.

## CONFLICT OF INTEREST STATEMENT

All authors have no conflicts of interest.

## Data Availability

The data that support the findings of this study are openly available.
